# 100th Anniversary of Jules Bordet's Nobel Prize: Tribute to a Founding Father of Immunology

**DOI:** 10.3389/fimmu.2019.02114

**Published:** 2019-09-11

**Authors:** Jean-Marc Cavaillon, Philippe Sansonetti, Michel Goldman

**Affiliations:** ^1^Institut Pasteur, Paris, France; ^2^Collège de France, Paris, France; ^3^Institute for Interdisciplinary Innovation in Healthcare (I3h), Université Libre de Bruxelles, Brussels, Belgium

**Keywords:** complement, serotherapy, anaphylatoxin, alexin, bacteriolysis, Metchnikoff, cattle pague, *Bordetella pertussis* (whooping cough)

## Abstract

The 100th Anniversary of the Nobel Prize in Physiology or Medicine 1919 awarded to Jules Bordet offers the opportunity to underline the contributions of this Belgian doctor to the blooming of immunology at the end of the nineteenth century at the Institut Pasteur de Paris. It is also the occasion to emphasize his achievements as director of the Institut Pasteur du Brabant and professor at the Université libre de Bruxelles. Both in France and Belgium, he developed a holistic vision of immunology as a science at the crossroads of chemistry, physiology, and microbiology.

## Introduction

Although the discovery of vaccination by Edward Jenner (1749–1823) at the end of the eighteen century was the first evidence for the existence of an immune system, immunology as a science only emerged more than one century later as a reflection of the host response to bacterial infections and bacterial toxins (1) with the pioneering works of Paul Ehrlich (1854–1915), Ilya Ilitch Metchnikoff (1845–1916), Emil von Behring (1854–1917), and Jules Bordet (1870–1961) (2, 3). Interestingly enough, two of these founding fathers of immunology worked at the Institut Pasteur de Paris, a unique scientific environment created in 1888 to build upon the seminal discovery of the anti-rabies vaccine by Louis Pasteur in 1885 (4).

**Figure 1:**
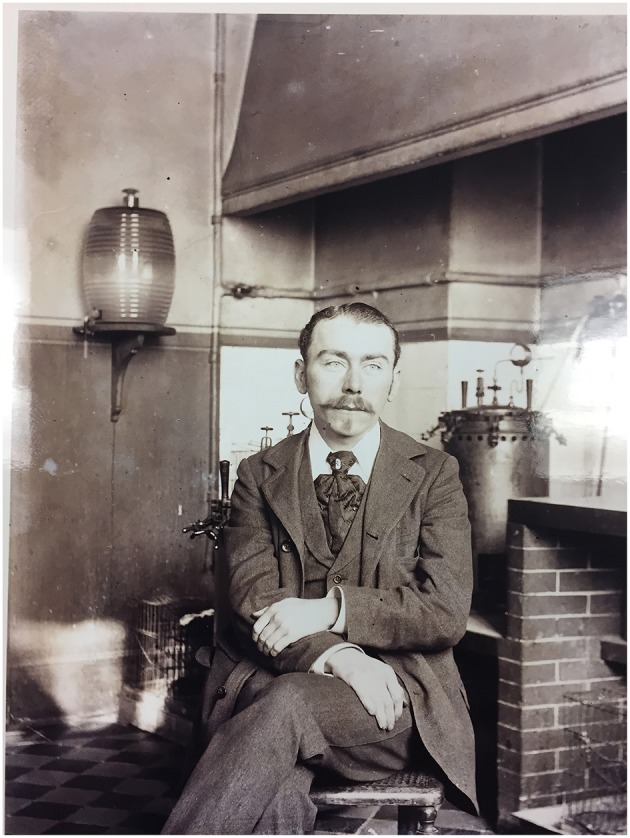
Jules Bordet in Elie Metchnikoff's laboratory.

Herein, we focus on Jules Bordet (Figure 1) as a scientist and a humanist, on the occasion of the 100th anniversary of his Nobel prize. We review the genesis of his major discoveries and conclude on Jules Bordet's legacy as a source of inspiration for future immunologists. On October, 28th, 1920, Jules Bordet was awarded with the 1919 Nobel prize, “for his discoveries relating to immunity,” namely his work on the complement system. He deciphered the mechanisms of the bacteriolytic activity of immune serum obtained in animals immunized with bacteria, and the hemolysis capacity of anti-red blood cell immune sera. But he was also a distinguished bacteriologist who worked on bacteriophages and discovered the causative bacterium of whooping cough, named *Bordetella pertussis*.

## The Early Years: From Botany to Immunology

Jules Bordet was born in 1870 in Soignies, a small Belgian town where his father Charles-Henri was a school teacher (5). At the age of 16, he entered the Université Libre de Bruxelles as a medical student, simultaneously with his older brother Charles. In parallel to their medical training, both brothers undertook experimental research. Jules Bordet was undoubtedly influenced by the ingenious studies of his brother on chemotactism (6, 7) when he started himself to investigate chemotactism of gametes of algae at the Botanical Institute of the Université libre de Bruxelles (8). It is in the same laboratory that he studied the mechanisms by which the virulence of *Vibrio metchnikovii* increased after serial passages in immunized guinea-pigs. He concluded from these experiments that the increased virulence was consecutive to a reduced toxicity and a reduced chemotactism. This work was published in 1892, in the Annales de l'Institut Pasteur with the following title “Adaptation des virus aux organismes vaccinés” (9). Jules Bordet obtained his medical graduation during the same year, 1 year ahead of his classmates. After a 1 year clinical experience in a hospital on the North Sea coast, he moved to the Institut Pasteur de Paris thanks to a travel award from the Belgian Government.

## A Founding Father of Immunology at the Institut Pasteur de Paris

**Figure 2:**
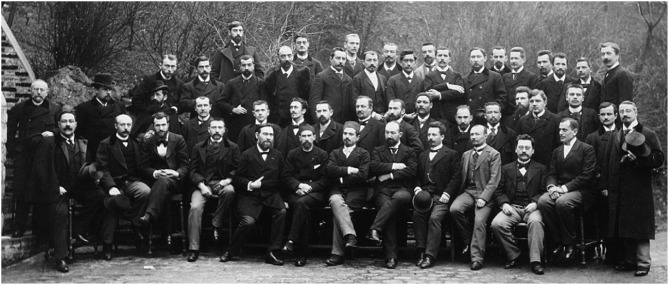
Group photo of the 1895 Technical Microbie Course of Institut Pasteur. First on the left, third raw: J. Bordet; Fifth and sixth from the left, first raw, seated: E. Metchnikoff and E. Roux. J. Danysz is standing just in front of J. Bordet.

Jules Bordet joined the Institut Pasteur in April 1894 where he attended the ≪ Grand Cours de Microbie ≫ organized by Dr. Emile Roux (Figure 2). This gave him the opportunity to meet Elie Metchnikoff, the father of cellular immunity. Very soon, Jules Bordet joined the Metchnikoff's laboratory in which he developed an independent line of research which culminated in the seminal demonstration that killing of bacteria depends on interactions between antigens, antibodies, and complement. Metchnikoff rapidly recognized the importance of Bordet's contributions which he already mentioned in his report to the International Congress of Budapest in 1894 (10). It is during the same period that Jules Bordet deciphered essential mechanisms of agglutination and lysis of sensitized red blood cells.

Jules Bordet stayed In Paris until 1901 with a 1 year interlude in 1897 when he conducted a scientific mission in the Transvaal (South Africa) on behalf of the Institut Pasteur. The objective was to study and possibly solve a rinderpest epidemic. He succeeded in this endeavor by devising a method based on a serotherapy principle. Serotherapy had been established by Emil von Behring and Shibasaburo Kitasato (11), and used on a large scale by Emile Roux (1853–1933) to cure diphtheria (12).

No doubt, that Bordet's stay at Institut Pasteur in the laboratory of Elie Metchnikoff, one of the most amazing and outstanding scientists of the time (13), deeply influenced the trajectory of his professional life. On the other hand, Jules Bordet remained a cherished fellow of the Institut Pasteur de Paris throughout his career as it was emphasized during the celebration of his 80th anniversary and during his funerals in 1961 (see Supplementary Materials).

## Back to Belgium to Lead a New Institut Pasteur

In 1901, Jules Bordet returned to Brussels where he had been invited to lead a new institute dedicated to anti-rabies vaccination and bacteriological studies. In 1908, this institute was eventually named *Institut Pasteur du Brabant* upon agreement by Louis Pasteur's widow. Jules Bordet's commitment to the management of the Institute did not prevent him to further develop his research activities. Indeed, this was the place where he developed pioneering serological tests based on complement fixation and engaged in successful bacteriological studies leading to the discovery of the whooping cough bacillus and the agent of avian diphtheria (5).

In 1907, Jules Bordet was appointed as the Chair of Bacteriology at the Université libre de Bruxelles where medical students enjoyed his inspiring courses on infectious agents and immune defenses. During the difficult times of World War I, Jules Bordet decided to take a focus his energy on an in-depth review of the knowledge available at the time on immune defenses. This endeavor resulted in a remarkable book entitled “Traité de l'immunité dans les maladies infectieuses,” which covered all the contemporary knowledge on immunology, illustrating his mastering of the field (14). After the war, Jules Bordet engaged himself in supporting the redevelopment of the Université libre de Bruxelles. It is during a fundraising mission in the United States in 1920 that he learned his designation as laureate of the 1919 Nobel Prize in Medicine or Physiology. In the ensuing years, Jules Bordet helped to establish a center for prophylaxis of the rinderpest in Brussels and became the President of the Conseil Scientifique de l'Institut Pasteur de Paris. He remained scientifically productive with a keen interest in bacteriophages (15–17). However, Bordet was the mastermind of the soluble enzyme hypothesis against the microorganism hypothesis (18). He transmitted his interest in bacteriophages to his son Paul Bordet (1906–1987) who succeeded him both at the Université libre de Bruxelles and at the Institut Pasteur du Brabant.

Jules Bordet infused his family with his passion for medicine and academic research across several generations. His grandchild André Govaerts (1930–2015), who became Chair of immunology at the Université libre de Bruxelles, demonstrated the involvement of cytotoxic lymphocytes in the rejection of renal allografts in dogs (19). In a touching testimony, his grand-daughter Raymonde Craps revealed the deep attention paid by Jules Bordet to his family (Supplementary Materials).

Toward the end of his life, the scientific curiosity of Jules Bordet remained insatiable, leading him to explore new territories such as astronomy, a topic on which he wrote a book (Elements d'astronomie, 1956) aimed to provide some basic clues to the visitors of the planetarium. Also, he became more and more concerned by philosophical, and societal aspects of science. In 1945, he published a booklet entitled “*Brèves considérations sur le mode de gouvernement, la liberté et l'éducation morale,“* in which he developed his broad vision of science and ethics as driving forces for all people of goodwill (20). Jules Bordet peacefully died in Brussels in April 1961.

## Major discoveries in perspective

### Identification of Complement and Its Interaction With Antibodies to Induce Bacteriolysis

Before Bordet published his first report on what we nowadays call the complement system, quite a few reports had already been published on the bactericidal activity of sera (Supplementary Figure 1, Supplementary Materials).

The seminal paper of Jules Bordet which led to a new concept of bacteriolysis was published in 1895 (21). It was his first article published during his stay in Metchnikoff's laboratory. It aimed at deciphering the so-called “Pfeiffer phenomenon,” namely the alteration and complete disintegration of *Vibrio cholerae* when introduced into the peritoneal cavity of an immunized guinea pig or into that of a normal one if immune serum is injected at the same time. For Metchnikoff, this observation was mainly due to the phagocytes, while Pfeiffer considered that it was the reflection of the bactericidal activity of the humors. Through an extensive series of experiments, Bordet demonstrated that heat-stable (56°C for 30 min) substances present in sera of immunized animals induced agglutination of vibrios but did not kill them. Indeed, the lysis of vibrios was found to require a heat-labile agent also present in the serum of unimmunized animals. These substances which were qualified as “sensitizers” correspond to the antibodies which were characterized later on. Bordet favored the word alexin, coined by Hans Buchner (1850-1902) to characterize the substance responsible for the bacteriolysis. Most interestingly, Bordet referred in his paper to the work of Joseph Denys (1857–1932) who had reported the same year, the process of opsonization (22). The word opsonization was coined later -in 1903- by Sir Edward Almroth Wright (1861–1947) (23). Bordet qualified as “stimuline” the positive action of so-called “preventive sera” to boost the phagocytosis process. However, for Metchnikoff, opsonization will never be a central concept to explain the most efficient collaborative role between phagocytes and serum factors. For example, he wrote in a preface of a book in 1915: “*The fact that phagocytosis is often “spontaneous”, independent from the contribution of opsonins, and the fact that researches on opsonic action have been performed in vitro, outside the body, does not allow to attribute a considerable role to this humoral factor*” (24). This comes a bit in contrast to the observation and the claims of Bordet.

Using more than 25 guinea-pigs, few rabbits and a goat, injected with different *cholera vibrios* strains and the *vibrio metchnikovii*, Bordet addressed the specificity and the cross-reactivity of the immune sera. Most importantly, he dissociated the preventive activity of the immune sera (antibodies) which provides the specificity from the heat-labile bactericidal activity (alexin/complement) present in both normal and immune serum, establishing the complementary activity of both components. For example, he reconstituted the bacteriolysis activity of a heat-treated preventive serum by adding fresh normal serum. But he also concluded that the bactericidal activity was derived from the leukocytes. This idea will be perpetuated in Metchnikoff's team, and while they were most probably observing the consequence of netosis discovered in 2004 (25), they maintained the idea that dead neutrophils contribute to the bactericidal activity found in sera (26). Indeed, the bacteriolytic activity of leukocyte lysates had been observed by many other investigators (27–29).

The following year Bordet published a paper entirely dedicated to phagocytosis, the specialty of his host laboratory (30). Working *in vivo* in guinea-pigs or *in vitro* with peritoneal exudates, he compared the phagocytic capacity toward an amazingly large panel of bacteria He reported that the speed, the intensity and the bactericidal activity varied from bacteria to bacteria. The same year, Bordet published a paper purely on humoral immunity, devoted to the mode of action of preventive sera (31). In this paper he wrote: “*We will have to quote many times the work of Mr. Metchnikoff, whose experience and valuable advice greatly benefit to those who work with him. May our dear and respected master receive here the expression of our gratitude.”* Interestingly, Bordet refers to the principle of agglutination, a word coined by Max von Gruber (1853–1927) in 1896 (32) concerning a mechanism already reported by Albert Charrin (1856–1907) in 1889 in Paris (33), which could be used for diagnosis. Interestingly, he also refers to hemagglutination (although the word had not yet been coined), a phenomenon similar to the agglutination of bacteria by immune sera. Referring to active and passive immunity, he supported the concept defended by Metchnikoff: “*Humors when they transform vibrios and alter them deeply, work, as repositories of active principles derived from leukocytes. If it is true that they can in some cases decimate the vibrios, however, phagocytosis always potently intervenes, acting by the end for the final destruction of the bacterium*.” While he considered that alexin is contained within the leukocytes and diffuses from them, he concluded that passive immunity increases the phagocytic bactericidal power.

In 1897, Bordet published an exhaustive analysis of the immunity against *Streptococcus* (34). He reported the extreme sensitivity of rabbits and the lower sensitivity of the guinea-pigs. Fascinatingly, Bordet described all the amazing properties of this bacterium, such as its capacity to prevent phagocytosis. A similar observation had already been made in Metchnikoff's laboratory when Nicolaï Tchistovitch (1860–1926) investigated the phagocytosis of *Pasteurella multicoda* by alveolar macrophages (35). Later on, it has been well-established that streptococcal M protein, H protein, and M-related protein contribute to the mechanism of evasion of phagocytosis by the bacterium. Furthermore, he mentioned that the bacterium had the capacity to survive within the phagocyte, a well-recognized pathogenic mechanism. He also reported the capacity of *Streptococcus* to interfere with the bactericidal activity of the sera, a function nowadays recognized as the reflection of certain streptococcal proteins to limit complement activation. Finally, he reported the major hemolysis observed during this type of infection, reminiscent of the capacity of this pathogen to release hemolysin. He also stated: “*Macrophages do not confine themselves to phagocyte streptococci on their own account, they also take in up more or less degenerate polymorphonuclear cells, which had previously seized the microbes*”. What Bordet was describing, nowadays known as efferocytosis, had actually already been described in Metchnikoff's laboratory by Marc Armand Ruffer (1859–1917) 7 years earlier (36, 37).

### Mechanisms of Hemagglutination and Hemolysis of Sensitized Erythrocytes

In the following years, Bordet focused his efforts on the phenomena of agglutination of red blood cells by antibodies and hemolysis by immune sera (38–40). He injected rabbit whole blood in the peritoneal cavity of guinea-pigs and found that immune sera from injected animals exert similar actions on the red blood cells as sera of animals immunized against vibrios. A heat-labile factor already present in normal serum was shown to contribute to hemolysis, while elements present in immune sera contributed to the agglutination of erythrocytes. It is much later that this process of hemagglutination was shown to depend on multivalent antibodies. Bordet also prepared immune sera against milk and showed their capacity to form agglomerates. He concluded that hemagglutination was a process similar to the process of coagulation. Preparing rabbit immune serum against chicken red blood cells, he showed the capacity of these sera to agglutinate and lyse the chicken erythrocytes, leaving alone the nucleus. *In vivo*, those were taken up by macrophages. Mixing rabbit blood with heat-deactivated (“decomplemented” as we would say nowadays) anti-rabbit erythrocyte serum from guinea-pigs, he showed that the rabbit alexin could contribute to the lysis of its own sensitized red blood cells. Similar demonstrations were made with rat, goat and dog sera as a source of alexin. The serum of guinea pigs was found a most efficient source of alexin whereas chicken alexin could not complement the activity of rabbit or guinea-pig anti-chicken erythrocytes. This led Bordet to propose that the alexin involved in bacteriolysis was the same than the alexin involved in hemolysis. He showed that the antibodies were directed against the stroma of red cells, since immunization with stroma of red blood cells ended to a similar hemolytic activity as when whole blood was used. Moreover, he observed that the anti-erythrocyte serum was lethal when injected intravenously in rabbits, whereas after subcutaneous injection he obtained an anti-hemolytic serum which could neutralize both the “sensitizers” and the alexin (41). He further reported that the generated anti-alexin, was species specific. Collectively, these studies of Jules Bordet establishing that red blood cells from different species could be recognized by antibodies in a specific manner, paved the way for the discovery of blood groups by Karl Landsteiner (1868–1943), another Nobel laureate (42).

### Serodiagnostic Tests Based on the Complement-Deviation Principle

Octave Gengou (1875–1957), a Belgian physician and bacteriologist, joined Bordet in Metchnikoff's laboratory. Marrying on July 1903, Berthe Levoz (1872–1941), the sister of Bordet's wife, he became his brother-in-law. Interestingly, in 1901, both reported that the antisera prepared against blood derived from different animal species, were also containing antibodies able to neutralize the coagulation process (43). The two Belgian partners also demonstrated that heat-inactivated immune sera raised against *Yersinia pestis, Bacillus anthracis, Erysipelothrix rhusiopathiae, Salmonella typhi, or Proteus vulgaris* could be bactericidal when supplemented with fresh guinea pig serum (44). Of note some of the immune sera were of horse origin, confirming that alexin can cross the species. A similar demonstration was performed with sera obtained from patients recovering from typhoid fever. Furthermore, Bordet and Gengou elegantly demonstrated the consumption of the alexin by a previous mixture of bacteria with the corresponding antiserum. When such a serum was mixed with a heat decomplemented mixture of red blood cells and anti-erythrocyte serum, no hemolysis could be observed, illustrating that the alexin had been absorbed by the first antigen-antibody complex. Such an approach has been exploited by August von Wassermann (1866–1925) and his German colleagues to develop a diagnosis test of syphilis (45), known as the Bordet-Wassermann test.

Bordet questioned the mechanism of interaction between the “sensitizers” and the alexin (46). In this paper, Bordet reasserted his attachment to the word alexin: “*It is therefore appropriate to give up with these names of zwischenkörper, amboreceptor, complement, terms which have been chosen under the influence of surely ingenious theoretical ideas, capable, thanks to the experiments they have inspired to allow progress of science, but for which the experience does not justify*”. For Bordet, once the antibodies bind their target, they allow the alexin to bind to this target, while his German competitors, Ehrlich and Morgenroth, favor a direct interaction of alexin with antibodies once the latter have interacted with their target (47). Nowadays, we know that indeed the complement system, upon activation by the classical pathway requires first the interaction of the C1 molecule with the antibody.

In a paper written, once back in Brussels together with Frederick Parker Gay (1874–1939), a visiting scientist from the USA, Bordet summarized his vision of the phenomenon as follows: “*Experimenters who studied hemolysis propose very different ideas about the relationships that are established between the sensitized globule and the active substances, sensitizer (amboceptor) and alexin (complement). It is well known, that first the globules bind the sensitizer (Ehrlich and Morgenroth), then the thus modified globules have acquired the power, which they did not display before, to absorb the alexin with such energy that they can strip it from the ambient liquid”* (48).

### Method for Rinderpest Control

Emil Adolf von Behring (1854–1917), first laureate of the Nobel Prize in physiology or medicine (1901), demonstrated in 1890 with Shibasaburo Kitasato (1853–1931) that passive immunization can treat or prevent tetanus (11), and reported with Erich Wernicke (1853–1931) that it could also protect against diphtheria (49), paving the way to serotherapy. Jules Bordet successfully applied serotherapy to rinderpest, also known as cattle plague. This devastating disease which killed close to 100% of affected animals, reached in 1896 the state of Transvaal in South Africa. The government of this free state decided to call upon European scientists to fight the disease. Thus, Robert Koch came from Berlin to Kimberley while Jules Bordet, accompanied by Jan Danysz (1860–1928), a Polish bacteriologist who had joined the Institut Pasteur, were sent for a scientific mission in Pretoria. On site, they received help from Arnold Theiler (1867–1936), a young Swiss veterinary surgeon who had an official position among the Transvaal authorities (50). Robert Koch advocated the injection of bile obtained from an infected ox, a treatment which eventually proved unsuccessful. Bordet and his colleagues focused their efforts on serotherapy. Collecting blood and sera from surviving infected animals, they could offer an efficient curative treatment (51). Ironically, the methods came to be known as “the method of the French doctors” (52). The expertise of Bordet acquired in Africa was useful when a rinderpest epizootic occurred in Belgium in 1920. Roux and Calmette went in Belgium to set up a program to fight the disease using immune sera, made both in Egypt and in the veterinary school of Cureghem, close to Brussels.

### Identification of the Agent of Whooping Cough

While in Paris, Bordet's 5 month-old baby girl got whooping-cough. Bordet discovered the presence of the responsible microorganism in her sputum, but could not isolated it until 1906, when his son, Paul, also got whooping-cough. Bordet and Gengou then developed an appropriate culture medium allowing them to isolate the bacillus. The medium prepared from potatoes, physiologic serum, agar, and rabbit blood was shown to also be convenient for other microorganisms (53). Bordet and Gengou specified that the bacterium was particularly abundant at the onset of the disease, then rapidly decreased in number. They also showed that the isolated microorganism could be lethal when used at high concentrations in guinea-pigs. Interestingly, they demonstrated that sera from convalescent children could agglutinate the bacteria and using the complement consumption approach confirmed that they contain specific antibodies. The anteriority of the work of our two Belgian doctors was disputed by Dr. Reyher, from the Charité Hospital in Berlin (54). Then Bordet and Gengou asked Reyher to send his isolated bacterium to allow a fine comparison. Reyher declined to do so. Bordet and Gengou thereafter presented a series of articulated arguments to demonstrate the inappropriateness of his claim (55). Their final scathing comment is worth to mention: “To conclude this long discussion, we feel that the columns of these Annals, our time, and certainly also that of Mr. Reyher, could have been more usefully employed.” The name *Bordetella pertussis* given to the pathogen unambiguously demonstrates that the scientific community gave full credit to Bordet for the discovery and identification of the microorganism. In 1909, Bordet and Gengou published their investigation on the endotoxin of *Bordetella pertussis* (56) and reported its strong toxicity in guinea pigs and rabbits and its lability to heat treatment. Knowing the heat resistance of endotoxins, and the specifically poor activity of *Bordetella pertussi*s endotoxin as compared to others (57), because its lipid A is penta-acylated (58), it is most probable that the Belgian scientists were dealing with a mixture of bacterial products rather than the purified endotoxin.

## Other Scientific Contributions

### Demonstration of Antibody-Independent Anaphylactoid Reactions

Bordet has been puzzled by the anaphylaxis phenomenon described by the French physiologist Charles Robert Richet (1850–1935) together with Paul Portier (1866–1962) (59). Richet was awarded the Nobel prize for his discovery in 1913. Bordet showed that agents other than antibodies can cause anaphylactic-like reactions; they were named anaphylatoxins. Together with Edward Zunz they demonstrated that agar added to guinea pig serum led to the formation of anaphylatoxin activity that was heat-sensitive (60). He therefore suggested that this activity was similar to alexin. Little attention was paid to this hypothesis until few decades later when complement activation was found to generate anaphylatoxins that induce the release of histamine from basophils (61).

### Identification of a Bacteriolytic Activity in Human Milk

In 1922, Alexander Fleming's (1881–1955) discovered that biological fluids can kill staphylococci *in vitro* (62). Subsequently, Bordet was the first to demonstrate that human colostrum and milk were bacteriolytic (63). He found that the causative agent was heat-resistant and therefore could not be related to complement. Actually, the bacteriolytic activity present in milk and other human secretory fluids was found five decades later to correspond to lysozyme.

## Concluding Remarks: Jules Bordet as a Continuous Source of Inspiration for Immunologists

One major lesson learned from Jules Bordet and which is still valid for today's scientists in biomedicine, particularly those at the onset of their career, is that doing basic research is fully compatible with a cross-disciplinary approach. The former route, thanks to in-depth investigation of selected biological processes, is essential to convincingly decipher vital principles that, in addition to decisively changing established views, also offers solid opportunities for game-changing applications in prevention and therapeutics. The latter route exploring neighboring—or sometimes remote—scientific territories and disciplines, broadens the scope of investigations and thereby offers a holistic perspective and increases chances to reveal the multiple facets of the studied topic. As a matter of fact, this is exactly how Jules Bordet proceeded. He could have focused on characterizing the nature and structure of antigens, or of antibodies, or on characterizing some alexin's components. He did not and decided to study the three in interaction, hence discovering the basic principle of serum bactericidal activity that illuminated the concept of humoral protection against infectious agents and offered entirely novel diagnostic methods and concepts to rationalize serotherapy. It is also by venturing more into microbiology which, at that time, was fully syncretic to immunology, that he discovered *Bordetella pertussis* thanks to a fruitful collaboration with Octave Gengou, a master in preparing complex growth media. By today's standards, evaluation panels might consider this as intellectual dispersion and would recommend to focus… What a nonsense it would have been! Why did Jules Bordet nonetheless succeed? Probably because his solid training in physiology helped him venturing in neighboring territories with a good compass often taking him to a pertinent destination. Indeed, broad initial education, developed sense of navigation in unknown territories, outstanding working capacity, may have been his recipe for success as a biomedical scientist. Furthermore, let's remember that many of the seminal contributions of Jules Bordet were based on studies using animal's or patients' specimens. In that sense, Jules Bordet can also be considered as a pioneer of translational research.

Exceptional individuals who successfully embraced several scientific or artistic disciplines are often qualified as polymaths (64). Jules Bordet is indeed recognized as a great polymath immunologist (5). More than ever, interdisciplinary approaches are needed to translate results from immunological research into medical advances. In the forthcoming era of precision medicine, system biology approaches will be required to integrate in-depth characterization of molecular signaling pathways together with knowledge on genetic, epigenetic, and environmental factors that shape immune responses. Novel clinical trial designs requiring careful ethical considerations, as well as innovative regulatory pathways and economic schemes will equally be important to make immune-based therapies and vaccines accessible and affordable. More than ever, polymath scientists with a holistic vision of immunology are needed (65). In order to educate and train them, academic institutions will have to break down silos and build upon the inspiring aphorism of Jules Bordet: “One of the great services that every science can render to our research is to invite us, as an introduction, to leave it for its neighbor” (14, translated from French).

## Author Contributions

All authors listed have made a substantial, direct and intellectual contribution to the work, and approved it for publication.

### Conflict of Interest Statement

The authors declare that the research was conducted in the absence of any commercial or financial relationships that could be construed as a potential conflict of interest.
