# Supplementary material for: 100th Anniversary of Jules Bordet's Nobel Prize: Tribute to a Founding Father of Immunology
Source: Front Immunol. 2019 Sep 11;10:2114. doi: 10.3389/fimmu.2019.02114 (PMC6749103; doi:10.3389/fimmu.2019.02114)
Supplement: Supplementary file 1 [file Data_Sheet_1.docx]

**Supplementary materials**

**Quotes from speeches of Pasteur Vallery-Radot and Alexander Fleming during the celebration of Jules Bordet’s 80th birthday anniversary**

**Professor Pasteur Vallery-Radot** alluded to the years spent by Bordet at Institut Pasteur as follows: “*Sir, at twenty-three, you entered the Institut Pasteur. You had blue eyes where the sky of your dreams beautifully externalized, a both malicious and benevolent smile, which attracted the sympathy of all, a singing and modulated intonation that reflected the kind country you came from and, under an apparent timidity, an assurance that made one says: "This little Jules knows what he wants, he will go far.*" […] “*Like Pascal, like Carnot, like Lavoisier, you were famous when you were thirty years old”.* Insisting on the influence of his Parisian masters who became his friend, the grand-son of Pasteur added: “*You have inherited the genius of Pasteur, the clarity of Roux, the elegance of Duclaux*” (10).

**Alexander Fleming** who was attending the celebration, also alluded to the blue eyes of Jules Bordet when he cited Paul de Kruif (1890-1971) an American microbiologist author of the book “Microbes hunters”: “*He had distracted eyes of a water blue, eyes that saw things that no one else was looking for seeing*.” Then, Fleming quoted Bordet himself to illustrate his modesty: “*In 1919, Bordet won the Nobel Prize. When congratulated of this honor in New York, he replied with his characteristic modesty: "I owe a lot to the French school of Pasteur who introduced me to scientific research ".*

**Quote from the speech of Jacques Tréfouël representing the Institut Pasteur during the funerals of Junes Bordet on 10^th^ April 1961**

“*It was in our institute, in Paris, that Bordet spent his very young years, near Metchnikoff, Besredka, his great friend, Monsieur Calmette, and his revered master Monsieur Roux. All of them had the illusion that this collaboration, because it was too fraternal, fruitful and invigorating, could be eternal, and it was not without heartbreak that they saw him leaving; the one whose place, left empty, was never completely replaced.* *A compensation was necessary to address this real sorrow, and Professor Bordet was able to fulfill the expectation. The Institute of Brabant, of which he was the founder and the soul for so long and fruitful years, he considered as a second Institut Pasteur, a name which Madame Pasteur offered him with a spontaneous joy, and it was in union as close as affective that the evolution of these two great Houses continued.”*

**Knowledges on bacteriolysis and hemolysis before Jules Bordet’s contributions**

Before Bordet published his first report on what we call nowadays the complement system, quite a few reports had already been published on the bactericidal activity of sera (**supplementary figure 1**).

In 1886, Josef von Fodor (1843–1901), a Hungarian doctor, two years before becoming the dean of the Faculty of Medicine of Budapest, reported the *in vivo* bactericidal activity of the blood, concluding that the organism was protected against the spread of bacteria by an unknown vital power of blood (1). The following year, he established *in vitro* that whole blood has the capacity to reduce the number of spiked anthrax bacilli (2).

In 1888, George Nuttall (1862–1937), an American-British medical doctor and bacteriologist was spending few years in the laboratory of Carl Flügge (1847-1923) in Göttingen. He was the first to demonstrate that defibrinated normal dog or rabbit blood possesses a considerable bactericidal power against anthrax bacilli, and that this power was destroyed by heating to 55°C for 30 min (3).

In 1889, Hans Buchner (1850-1902), a German physician and bacteriologist working in Münich, confirmed the previous observations on the ability of cell-free serum to destroy typhoid bacilli and on its thermolability (4). And in lecture, held at the Medical Associations in Munich (June 3, 1891) he named “alexine”, from Greek meaning “to ward off”, the substance capable to destroy bacteria (5,6). In 1893, Buchner described the mechanism of hemolysis (7).

The word “alexin” was immediately criticized by Hankin: “*Since I am the first discoverer of protective albuminous bodies, I have the right to give their nomenclature. For one and a half years, these bodies have been called "protective albumen" and in my opinion there is no sufficient reason to change that name*" (8). The same dispute occurred at the 7^th^ International congress of Hygiene, and Demography in London (10-17 August, 1891), when Hankin stated that the new name could give rise to confusion.

Indeed, in 1890 Ernest Hanbury Hankin (1865 – 1939) had identified a “bacterial killing globulin” (9). Hankin is a famous English bacteriologist who inoculated himself with cholera bacillus together with Waldemar Haffkin (1860-1930), his colleague from Institut Pasteur and some other enthusiastic scientists (10). He worked in India on cholera, malaria and reported the presence of bacteriophages in the waters of the Ganga. He appropriately described the complement consumption: “*A curious point about this property of blood serum is that it vanishes in the act of killing the microbes. That is to say, a given quantity of blood serum can only kill a limited number of microbes. If the number of microbes added to the serum is beyond this limit, the survivors find the blood serum to be an excellent food medium, and, after a time, begin to grow and reproduce*.” But his work was also performed on extracts from lymphatic glands of cats or dogs, which displayed a similar bactericidal activity as the serum. He ended to the conclusion that: “*phagocytes possess a substance having bacteria-killing powers, and we may suppose that phagocytes can not only kill* *microbes that they have ingested, but also do this, or tend to do this, by breaking down and liberating their contents.”* In 1892, Hankin, ended his fight against the word alexine since he entitled his new paper “*Report on the bactericidal action of alexines*” (11). Again, in this work he concomitantly addressed the bactericidal “power” of sera and of extracts from dog and rabbit spleens.

Richard Pfeiffer (1858 –1945) is a German physician and bacteriologist, who coined the concept of endotoxin (12). In 1894, he described the *in vivo* phenomenon of bacteriolysis in conjunction with vaccination (13). *Cholera vibrios* introduced into the peritoneal cavity of a guinea pig that has been immunized against cholera, loose their motility, disintegrate and pass into the solution (granular degeneration). The disintegration could be followed under the microscope by removing a portion of the peritoneal contents from time to time. The same result was observed if a bacteriolytic serum against cholera was introduced along with the bacteria into the peritoneal cavity of a non-immunized guinea pig. This observation become renown as the “Pfeiffer phenomenon”.

Still in 1894, a Belgian doctor, Joseph Denys (1857 - 1932) compared the bactericidal activity of dog whole blood and that of serum. He did it also with human, hen and pigeon blood. He observed that the bactericidal power of blood was far greater than that of serum. He came to the conclusion that: “*Neither the phagocytic theory nor the humoral theory, taken separately can explain the immunity. The phagocytes and the humors contribute together, to a varying degree according to the species, and also probably according to the nature of the aggressor, to preserve the higher organisms against the invading microbes*” (14). Indeed, the confusion has been regularly perpetuated by scientists who were associating the bactericidal activity displayed by serum and that found in leukocyte lysates (15). This will be also the case with Jules Bordet, under the influence of his mentor.

Paul Ehrlich (1854 – 1915), is a German physician and scientist, who is considered as the father of the humoral immunity with his famous side-chain theory of antigen–antibody interaction. He shared with Metchnikoff the Nobel prize in 1908. In 1899, concomitantly with Bordet’s investigations, Ehrlich coined the word “complement” to characterize this complementary activity, present in sera, which allows the antibodies to end to bacteriolysis (16,17). He also favored the idea that the complement was first interacting with the antibodies. The word of complement will become the most popular one, even if his book published in 1920 (18), Bordet still mainly talk about alexine, considering that the term “complement” was not justified: *"As for the lytic agent, Buchner, in his interesting researches on the bactericidal or globulicidal power of normal serums, had called it with the elegant name of alexine. The term therefore has a priority right. When Bordet recognized that the same principle played a considerable role in the lytic activity of antisera, it appeared to him that logic and propriety forbade changing the name. It also has the advantage of being exclusive of any interpretation as to the mode of reaction of the active substances."* May be, his choice also reflects a long controversial relationship with Ehrlich. Amusingly, during the award ceremony speech for the Nobel prize on December 10, 1920 (which was not attended by Bordet, since he was touring in USA), Professor A. Petterson, member of the Staff of the Professors of the Royal Karolinska Institute stated about alexine: “*It is also called by the more usual name of “complement*”. And he used nine times the word “complement” and only four times for the word alexine.

**REFERENCES**

1. Fodor J. Bakterien im blute lebender thiere. Arch Hyg (1886), 4:129-148
2. Fodor J. Die fahigkeit des blutes bakterien zu vernichten. Dtsch Med Woch (1887) 8: 745-747
3. Nuttall G. Experimente uber die bacterienfeindlichen einfluesse des thierischen korpers Z. Hyg Infektkr. (1888) 4, 353-94
4. Buchner H. Ueber die bakterientödtende Wirkung des zellenfreien Blutserums. Zbl. Bakt., Parasitol (1889) 5, 817–23; 6, 1–11
5. Buchner H. Kurze Uebersicht über die Entwicklung der Bakterienforschung seitNaegeli’s Eingreifen in dieselbe. Vortrag, gehalten im Aerztlichen Vereine in München am 3. Juni 1891. Münchener medizinische Wochenschrift. 1891. No. 25 (S435) & 26 (S454.
6. Buchner H. Zur Nomenklatur der schutzenden Eiweisskorper. Centr Bakteriol Parasitenk. (1891) 10: 699–701.
7. Buchner, H. (1893) Deber den einfluss des lichtes auf bacterien und uber die selbstreinigung der Flusse. Arch. Hyg. Berl. 17, 179-201*. 1893.
8. Hankin EH. Ueber die nomenclatur der schützenden eiweisskörper. Zbl. Bakt., Parasitol (1891), 10, 377-79.
9. Hankin. A bacterial killing globulin. Proc. Royal. Soc. London (1890) 48, 93-101
10. Cholera Inoculation. The Hospital (1892) 12 :401-2.
11. Hankin EH Report on the bactericidal action of alexins Brit Med J (1892), 2(1657): 728–730.
12. Rietschel ET, Cavaillon JM. Richard Pfeiffer and Alexandre Besredka : creator of the concept of endotoxin and anti-endotoxin. Microbes and Infection (2003) 5, 1407-1414
13. Pfeiffer R. Weitere untersuchungen über das wesen der choleraimmunität und über specifische processe Z. Hyg. Infektkrabkh (1894) 18: 1-16
14. Denys J, Havet J. Sur la part des leucocytes dans le pouvoir bactéricide du sang de chien. La cellule (1894) 10 : 7-35.
15. Schattenfroh A. Ueber die backerienfeindlichen Eigenschaften der Leukocyten. Arch. Hyg. (1897) 31, 1-81.
16. Ehrlich P, Morgenroth J. Ueber Haemolysine : zweite Mittheilung. *Berliner klinische Wochenschrift* (1899) 36: 481-486.
17. Ehrlich P, Sachs H. Ueber die Vielheit der Complemente des Serums. *Berliner klinische Wochenschrift* (1902) 39: 297-299 und 335-338.
18. Bordet J. Traité de l’immunité dans les maladies infectieuses. (1920) Masson et Cie Editeurs.

**
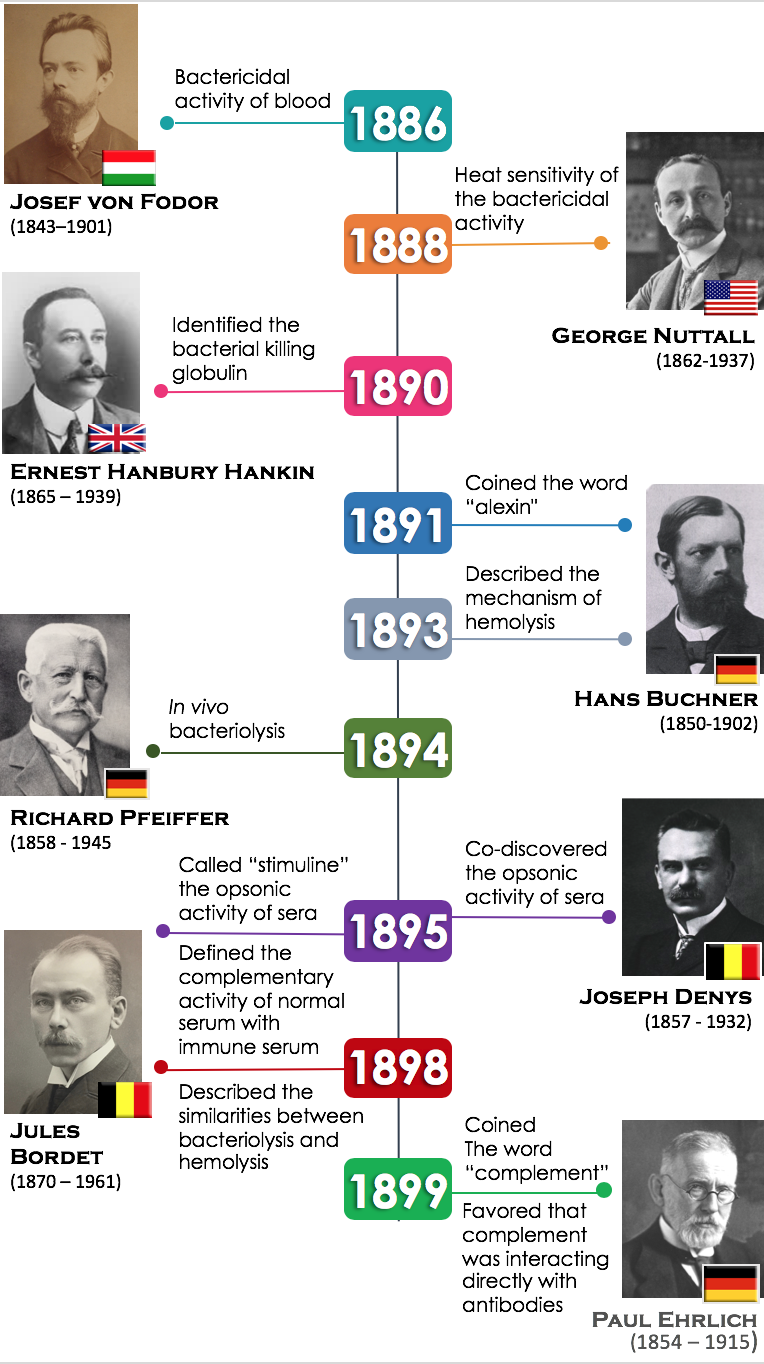
**

**Legend to supplementary figure 1**. Main steps in the discoveries of the complement system by the end of the XIXth century
